# Physician assistants/associates in psychiatry: a workforce analysis

**DOI:** 10.1186/s12960-024-00911-2

**Published:** 2024-06-18

**Authors:** Mirela Bruza-Augatis, Andrzej Kozikowski, Roderick S. Hooker, Kasey Puckett

**Affiliations:** 1 National Commission on Certification of Physician Assistants, 12000 Findley Road, Suite 200, Johns Creek, GA 30097 USA; 2 Retired Health Services Research, Ridgefield, WA USA

**Keywords:** Psychiatry, Behavioral health, Physician associates, Physician assistants, Workforce, Employment

## Abstract

**Background:**

Physician assistants/associates (PAs) provide services in diverse medical specialties globally, including psychiatry. While health professionals in psychiatry have been described for many years, little is known about PAs practicing in this discipline.

**Methods:**

We describe US PAs practicing in psychiatry using robust national data from the National Commission on Certification of Physician Assistants (NCCPA). Analyses included descriptive and inferential statistics comparing PAs in psychiatry to PAs in all other medical and surgical specialties.

**Results:**

The percentage of PAs practicing in psychiatry has increased from 1.1% (*n* = 630) in 2013 to 2.0% (*n* = 2 262) in 2021. PAs in psychiatry differed from PAs practicing in all other specialties in the following: they identified predominately as female (71.4% vs. 69.1%; *p* = 0.016), were more racially diverse (Asian [6.6% vs. 6.0%], Black/African American [5.5% vs. 3.4%], multi-race [2.8% vs. 2.1%], and other races [Native Hawaiian/Pacific Islander, American Indian/Alaska Native, or other; 3.7% vs. 3.6%]; *p* < 0.001), and resided in the South (43.8% vs. 34.1%; *p* < 0.001). PAs in psychiatry vs. all other specialties were more likely to work in office-based private practice settings (41.6% vs. 37.3%; *p* < 0.001) and nearly twice as likely to provide telemedicine services for their patients (62.7% vs. 32.9%; *p* < 0.001). While one-third (31.9%) of PAs in psychiatry experienced one or more burnout symptoms, and 8.1% considered changing their current position, the vast majority of PAs in psychiatry (86.0%) were satisfied with their position.

**Conclusions:**

Understanding the attributes of PAs in psychiatry is essential in medical labor supply and demand research. Our findings suggest that the number of PAs working in psychiatry is steadily increasing. These PAs were predominantly female, exhibited greater racial diversity, and were primarily located in the South and Midwest regions of the US. A striking difference was that PAs in psychiatry were almost twice as likely to provide telemedicine services for their patients. Although nearly a third of PAs in psychiatry acknowledged having one or more symptoms of burnout, few were considering changing their employment, and the vast majority reported high job satisfaction.

## Introduction

According to the Global Burden of Disease study, in 2019, one in every eight persons worldwide suffered from a mental health disorder [1]. This figure increased to nearly a third at the onset of the COVID-19 pandemic [2]. To promote mental health for all, the World Health Organization (WHO) expanded the *Comprehensive Mental Health Action Plan*; however, to realize this goal, there is a need for increased mental health resources—particularly a sufficient and capable mental health workforce to meet the population’s needs [3]. There was a slight increase in the median number of mental health care workers, such as psychiatrists and social workers globally—from 9 to 13 per 100 000 people between 2014 and 2020. However, despite this increase, projections suggest that the demand for mental health services will surpass the capacity of the current supply of providers [4].

In the United States (US), statistical trends reveal a concerning picture regarding mental health. The National Institute of Health (NIH) reported that in 2021, 57.8 million adults suffered from a mental health disorder, and less than half (47.2% or 26.5 million) received treatment [5]. Despite the US government allocating approximately $280 billion of federal funds for mental health services in 2020, a significant gap persists in ensuring access—about a third (29%) of individuals reported not knowing where to seek mental health services [5].

The situation will likely worsen due to predicted shortages of psychiatrists in the US [6]. Research by Satiani and colleagues, utilizing three national data sources, forecasts a deficit of between 10 538 and 28 521 psychiatrists by 2030 [7]. A study by Beck et al. indicated that there were 66,740 mental healthcare professionals, including 47 046 psychiatrists, 17 534 psychiatric nurses and nurse practitioners^1^ (NPs), 1,164 PAs, and 966 psychiatric pharmacists [11]. The authors determined that there were only 2.1 psychiatric providers per million people, which is insufficient for the mental health care needs in the US [11]. Furthermore, not all mental healthcare professionals can provide the same services; in the US, only physicians, NPs, and PAs are licensed to initiate prescriptions. These estimates were calculated before the COVID-19 pandemic, which has exacerbated the situation by causing a surge in anxiety, depression, and substance use disorders [2]. This increase in mental health disorders, combined with predicted shortages in the psychiatry workforce, poses a significant barrier to accessing mental health services and is a pressing public health concern nationwide [12].

PAs are recognized as an important strategy to help address the growing demand for psychiatric care in the US and worldwide [13, 14]. Founded in the 1960s, PAs are trained in the same medical model of care as physicians, and their education is focused on a primary care curriculum [15]. The criteria for employment as a PA are to graduate from an accredited graduate-level education program, pass a national examination to be board certified, and be licensed by a state or territory of the US [16]. The profession voted to change the name from “physician assistant” to “physician associate” in 2021; both are used interchangeably. This modification is consistent with the PA (physician associate) name in the United Kingdom, Ireland, Australia, and New Zealand [17]. PA and 'PA comparable’ professions (e.g., feldsher, clinical officer, etc.) have existed since the eighteenth century in France, Puerto Rico, and Russia [18]. They are found in over 50 countries globally [19] and provide care in various medical disciplines. Many PA/PA comparable providers report working in primary care settings and predominately providing services in mental health, maternal care, and surgical care [20–22]. Although some differences have been identified in their roles within the healthcare systems in their respective countries, there is ongoing research to examine the scope of practice of PA/PA comparable professions worldwide [17].

In the US, PAs in psychiatry work in collaboration with psychiatrists in team-delivered care and are in demand in this specialty [23]. The first mention of the utilization of PAs for mental health services dates back to 1984 at a Nebraska state hospital, highlighting their contributions [24]. More recent literature recognizes the significant role of PAs in psychiatry, particularly in enhancing access and improving the quality of psychiatric care [25]. Stefos observed that PAs significantly improved the productivity of psychiatrists within the mental health workforce in the US Department of Veteran Affairs (VA) facilities [26]. Curran and colleagues interviewed employees in inpatient psychiatric institutions in the US [27]. The authors found that facilities employing PAs reported high satisfaction and quality patient care and decreased physician workload [27]. In a separate study, Curran et al. conducted interviews examining the scope of practice of PAs in psychiatric care [27]. The researchers discovered that PAs performed a wide range of activities, such as taking history and conducting physicals, diagnosing and treating patients with psychiatric conditions and substance use disorders [27]. More recently, in 2020, Mauldin et al. examined the role of PAs in providing mental health care and discovered that 62% of PAs across all specialties evaluated people for mental health symptoms weekly [28].

The rising prevalence of behavioral health issues, coupled with the shortage of psychiatrists and the imperative need for additional mental health providers, underscores the need for further research into the PA workforce. Existing literature offers limited insight into the attributes of PAs in psychiatry and how they differ compared to PAs practicing in other specialties. Moreover, no prior study used national datasets to explore the characteristics of PAs practicing in the psychiatry discipline. For these reasons, we undertook a study to examine the demographics and practice characteristics of PAs working in psychiatric roles versus those in all other medical and surgical specialties. The aim is to identify the unique qualities of PAs in psychiatry and how they differ from their peers in other medical and surgical environments. Understanding these distinctions is essential for effective workforce planning and resource allocation. The objective of this census is to assist strategies underway addressing healthcare workforce disparities in psychiatry and to better meet society’s needs [29].

## Methods

We analyzed comprehensive PA workforce data from the National Commission on the Certification of Physician Assistants (NCCPA). NCCPA ensures data quality by following quality assurance protocols and ensuring PA anonymity. In addition to administrative information, the NCCPA collects self-reported demographic and practice data through the *PA Professional Profile* on board certified PAs in the US [30]. The *PA Professional Profile* consists of workforce questions initially launched in 2012 and supplemented with new items based on a comprehensive literature review each year. This instrument was conceived in alignment with the minimum dataset (MDS) guidelines of the Health Resources and Services Administration (HRSA) and in consultation with other organizations [31–34]. The MDS recommends collecting foundational workforce information related to different health professions' education/training, demographics, and practice characteristics that can help better understand the supply and distribution of the health workforce. This information supports more accurate and effective health workforce planning efforts [34].

Our study's inclusion criteria required participants to have updated their *PA Professional Profile* within the last three years, confirmed their clinical practice, and identified the specialty of their principal clinical position. Of the total number of board certified PAs at the end of 2021 (*n* = 158 470), 133 903 PAs (84.5%) had updated at least a portion of the *PA Professional Profile* within the last three years. To determine if there were differences between PAs who updated their information vs. those who did not, we used administrative data, which had less than 0.01% missing data. The results showed no meaningful differences between the two groups in terms of gender (70.1% vs. 70.3% female), age group (38.1% vs. 39.7% aged 30–39 years), US region (34.6% vs. 33.8% residing in the South), and rural–urban setting (92.7% vs. 94.5% urban). Of the 133 903 PAs who updated their information, 88.9% (or 119 010 PAs) responded to the question about practicing clinically. Of these, 93.9% (111 724 PAs) confirmed they were practicing clinically, while 6.1% (or 7 286 PAs) said they were not. Among the PAs who confirmed they were practicing clinically, 99.7% (or 111 428 PAs) provided their specialty. This group formed our analytical sample.

For our primary analysis using 2021 data, we compared the number of PAs in psychiatry (*n* = 2 262) with the total of those in all other specialties (*n* = 109 166) through their distribution, demographics, practice characteristics, and other important attributes (e.g., income, job satisfaction, burnout, intention to leave their current clinical position in the next 12 months, and intention to retire in the next five years). We calculated descriptive statistics and conducted bivariate analyses (e.g., Chi-square, Mann–Whitney) for all variables to compare PAs practicing in psychiatry to PAs in all other specialties. To determine trends in the number and proportion of PAs in psychiatry, we assessed nine years of data (2013 to 2021). For all analyses where a comparison is made, a *P* value of 0.05 or less is considered statistically significant. This research was exempted by the Sterling Institutional Review Board (IRB# 8759), and analyses were computed using SPSS version 28.

## Results

**Fig. 1:**
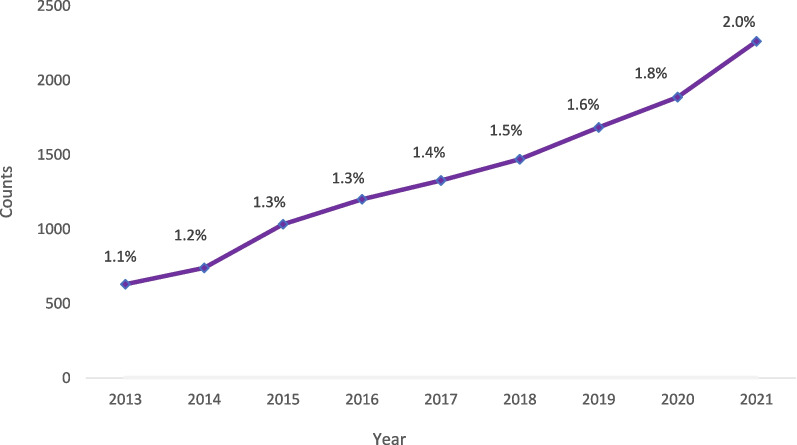
Growth of PAs in psychiatry from 2013 to 2021

**Fig. 2:**
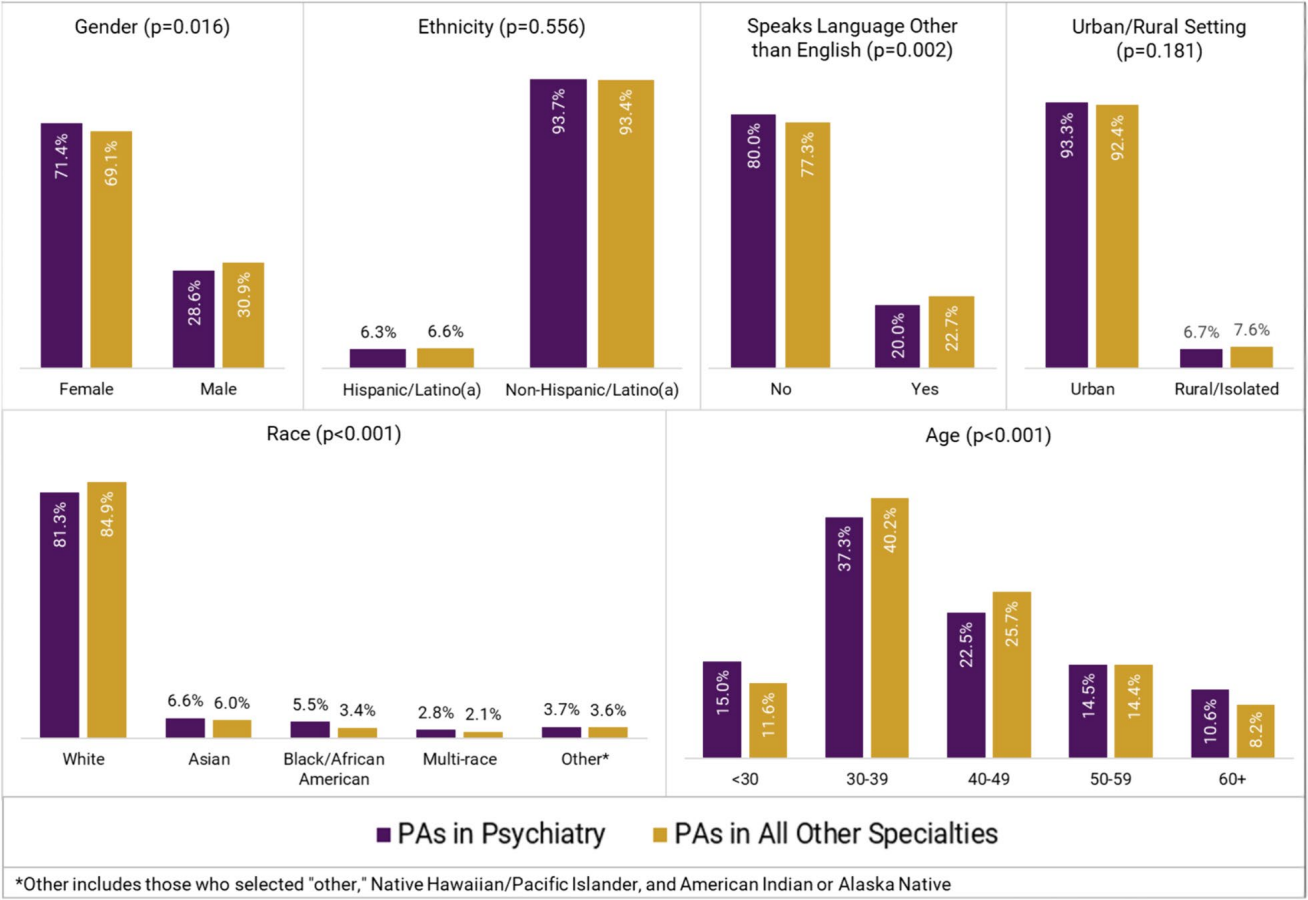
Demographic breakdown of PAs in psychiatry vs. all other specialties

The percentage of PAs practicing in psychiatry increased from 1.1% (*n* = 630) in 2013 to 2.0% (*n* = 2 262) in 2021 (Fig. 1). Our results revealed that in 2021, the PA psychiatric workforce was predominately female (71.4% vs. 69.1%; p = 0.016), younger than 30 years old (15.0% vs. 11.6%; *p* < 0.001), and more racially diverse (Asian [6.6% vs. 6.0%], Black/African American [5.5% vs. 3.4%], multi-race [2.8% vs. 2.1%], and other races [Native Hawaiian/Pacific Islander, American Indian/Alaska Native, or other; 3.7% vs. 3.6%]; *p* < 0.001) than the remainder of the PA workforce (Fig. 2). However, PAs in psychiatry were less likely to speak a language other than English with their patients (20.0% vs. 22.7%; *p* = 0.002) than PAs in all other specialties. No statistically significant differences were detected in terms of ethnicity (*p* = 0.556) and urban–rural setting (*p* = 0.181) (Fig. 2). However, there was a significant difference by US region (*p* < 0.001); a higher proportion of PAs in psychiatry vs. PAs in all other specialties worked in the South (43.8% vs. 34.1%) and the Midwest (22.1% vs. 19.8%).

The principal practice setting of PAs employed in psychiatry differed substantially from all PAs (*p* < 0.001). Compared to PAs in all other specialties, PAs in psychiatry worked predominantly in office-based private practice (41.6% vs. 37.3%) and federal government facilities (7.5% vs. 4.8%). For both groups, the median number of years certified differed somewhat (7 [PAs in psychiatry] vs. 10 [PAs in all other specialties]; *p* < 0.001). Most noteworthy, PAs practicing in psychiatry were almost twice as likely to provide telemedicine services compared to PAs in all other disciplines (62.7% vs. 32.9%; *p* < 0.001) and spent more hours using telemedicine than their counterparts (*p* < 0.001). Moreover, PAs in psychiatry vs. PAs in all other specialties worked 31 to 40 h a week (66.1% vs. 56.1%; *p* < 0.001) and reported seeing, on average, slightly fewer patients weekly (63.0 vs. 67.6; *p* < 0.001) (Table 1).

**Table 1:** Practice characteristics of PAs in psychiatry vs. PAs in all other specialties

	PAs in psychiatry (*N* = 2 262)		PAs in all other specialties (*N* = 109, 166)	
	Number	Percent (%)	Number	Percent (%)
Practice setting (*P*-value < 0.001)				
Office-based private practice	940	41.6	40,697	37.3
Hospital	399	17.7	45,881	42.1
Federal government	169	7.5	5272	4.8
Other	749	33.2	17,178	15.8
**Total**	**2 257**	**100.0**	**109, 028**	**100.0**
US region (*P*-value < 0.001)				
South	990	43.8	37, 144	34.1
Midwest	499	22.1	21, 515	19.8
Northeast	387	17.1	27, 173	25.0
West	382	16.9	22, 966	21.1
**Total**	**2 263**	**100.0**	**108, 798**	**100.0**
Secondary job (*P*-value = 0.014)				
No, I work in only one clinical position	1 875	83.3	92, 252	84.8
Yes, I also work in a position where I do not provide direct patient care (i.e., education, research, administration)	77	3.4	4 183	3.8
Yes, I work in two or more clinical PA positions	298	13.2	12, 337	11.3
**Total**	**2 250**	**100.0**	**108, 772**	**100.0**
Hours worked per week (*P*-value < 0.001)				
Up to 30	292	12.9	14, 357	13.2
31–40	1 495	66.1	61, 286	56.1
41–50	388	17.2	26, 376	24.2
51 +	87	3.8	7 132	6.5
**Total**	**2262**	**100.0**	**109, 151**	**100.0**
Mean and median of hours worked per week (*P*-value < 0.001)				
Mean (SD)* Median (IQR)**	39.2 (9.0) 40.0 (40.0–40.0)		40.0 (10.4) 40.0 (36.0–45.0)	
Patients seen each week (*P*-value < 0.001)				
Up to 40	683	30.2	31, 252	28.7
41–60	647	28.6	27, 713	25.4
61–80	419	18.5	20, 586	18.9
81–100	318	14.1	16, 562	15.2
101 +	194	8.6	12, 967	11.9
**Total**	**2 261**	**100.0**	**109, 080**	**100.0**
Mean and median of patients seen each week (*P*-value < 0.001)				
Mean (SD)* Median (IQR)**	63.0 (34.4) 60.0 (40.0–80.0)		67.6 (43.2) 60.0 (40.0–90.0)	
Participate in telemedicine (*P*-value < 0.001)				
No	842	37.3	72, 868	67.1
Yes	1 413	62.7	35, 796	32.9
**Total**	**2 255**	**100.0**	**108, 664**	**100.0**
Hours in telemedicine (*P*-value < 0.001)				
< 10	443	31.4	27, 940	78.1
10–19	237	16.8	4 732	13.2
20–29	251	17.8	1 906	5.3
30–39	264	18.7	741	2.1
40 +	217	15.4	458	1.3
**Total**	**1 412**	**100.0**	**35, 777**	**100.0**
Years certified (*P*-value < 0.001)				
Up to 10	1 408	62.2	56, 278	51.6
11–20	508	22.5	33, 868	31.0
21 +	346	15.3	19, 020	17.4
**Total**	**2 262**	**100.0**	**109, 166**	**100.0**
Mean and median of years certified (*P*-value < 0.001)				
Mean (SD)* Median (IQR)**	10.6 (9.4) 7.0 (3.0–16.0)		12.2 (8.8) 10.0 (5.0–18.0)	

*SD, standard deviation; **IQR, interquartile range

**Fig. 3:**
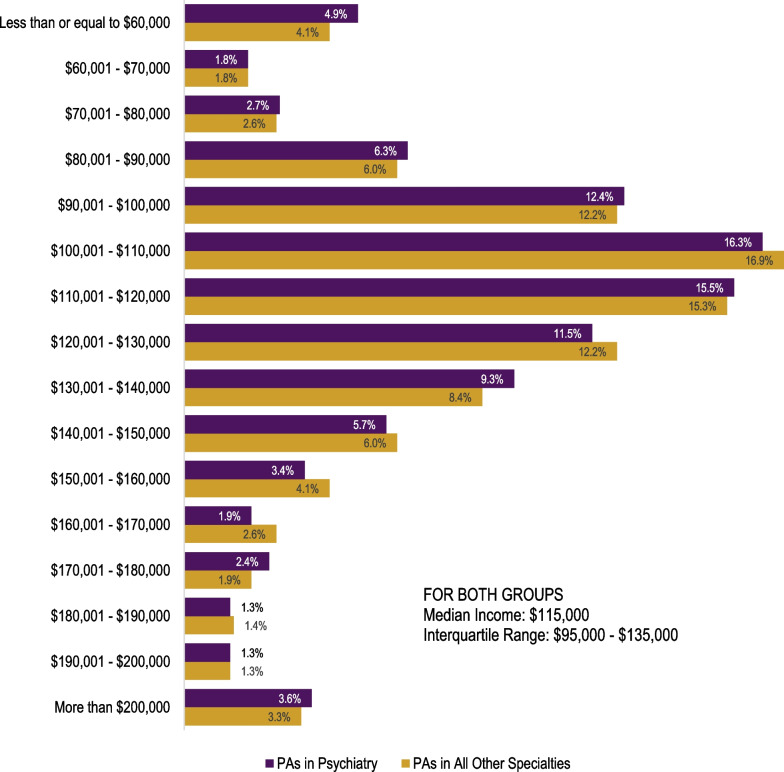
Income brackets of PAs in psychiatry vs. PAs in all other specialties (*p* = 0.267)

**Fig. 4:**
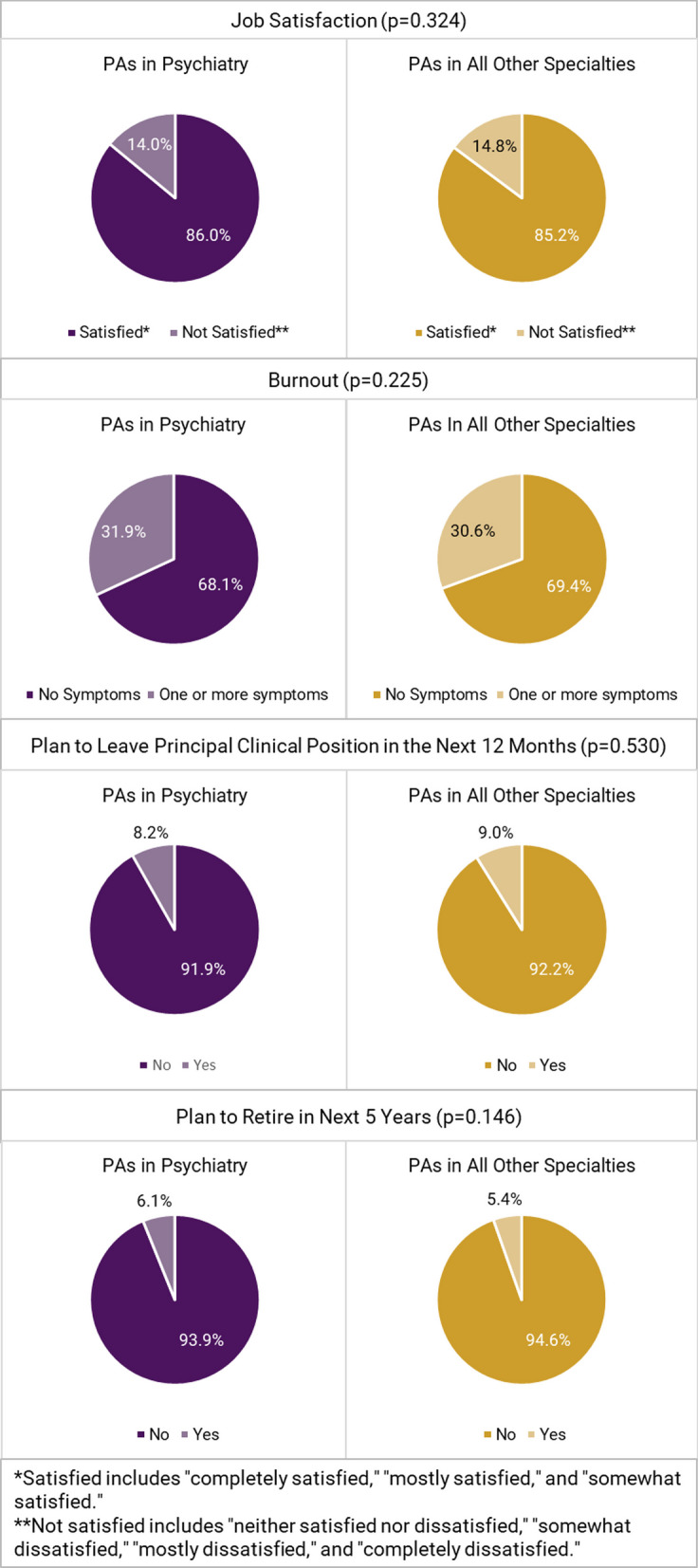
Job satisfaction, burnout and career plans of PAs in psychiatry vs all other specialties

The self-reported income distribution of PAs was the same for both groups (*p* = 0.267), with a reported median of $115 000 and an interquartile range of $95 000 to $135 000 (Fig. 3). The likelihood of reporting one or more symptoms of burnout (31.9% vs. 30.6%; *p* = 0.225), intending to leave their principal clinical position within the next 12 months (8.1% vs. 7.8%; *p* = 0.530), and job satisfaction (86.0% vs. 85.2%; *p* = 0.324) were all not statistically significantly different between PAs in psychiatry and those in all other specialties (Fig. 4).

## Discussion

Our study revealed several key characteristics distinguishing PAs in psychiatry from those in other fields. PAs in psychiatry were predominantly female, exhibited greater racial diversity, and were primarily located in the South and Midwest regions of the US. A striking finding was that PAs in psychiatry were almost twice as likely to provide telemedicine services for their patients. Although nearly a third of PAs in psychiatry acknowledged having one or more symptoms of burnout, few were considering changing their employment, and the vast majority reported high job satisfaction.

Our findings are consistent with but also provide an update to those of Ginther, Woyziak, and Quigley’s research conducted in 2009. The researchers surveyed PAs in psychiatry and found that 46% worked in private clinics or hospitals [35]. Our study using national data from the end of 2021 showed that PAs in psychiatry worked primarily in office-based private practice. The US Bureau of Labor Statistics (BLS) reports that as of 2022, most psychiatrists provided care in outpatient centers or office-based practices [36]. We also found that nearly two-thirds of PAs in psychiatry work in the US South and Midwest. These two US regions have the least psychiatrists [37]. Moreover, research shows over half of all US counties do not have a psychiatrist—the shortage in rural counties is even more severe [37]. We found that 6.7% of PAs in psychiatry practice in this setting. These findings suggest that PAs in psychiatry play a vital role in helping to fill gaps in care in rural and underresourced areas.

One of the most important findings observed in our study was the high proportion of participation in telemedicine/telehealth among PAs in psychiatry. We found that roughly two-thirds of PAs in psychiatry utilized telemedicine to diagnose, treat, and manage patients with mental health disorders. The adoption of telemedicine/telehealth has been growing, and data suggest that usage rates in the field of psychiatry have surpassed other specialties [38]. Even in the pre-COVID-19 era, a higher proportion of psychiatrists than other physicians relied on telemedicine to care for their patients [39]. This trend underscores the growing importance and potential role of telemedicine technology to extend both psychiatrists' and PAs' reach to individuals in need of mental health care.

Furthermore, our research discovered that although about one-third of PAs in psychiatry indicated burnout symptoms, less than one-tenth considered changing their employment, and the majority continued to report high job satisfaction. Compared to other healthcare professions, a smaller proportion of PAs in psychiatry reported intending to change jobs and having burnout symptoms. Summers et al. found that nearly two-thirds of psychiatrists in North America indicated high levels of burnout [40]. A recent study noted that 50% of physicians and 25% of US nurses have considered leaving their current employment in 2023 [41]. Dyrbye and colleagues found that over one-third (38.5%) of PAs and NPs indicated at least one symptom of burnout [42]. The same study also reported that nearly one-third of PAs/NPs indicated the intention to leave their clinical employment within the year [42], a significantly higher percentage than observed in our study. However, a potential reason for the difference is that Dyrbye et al. did not separate NPs from PAs in their analysis. Similar results have been reported globally. A 2023 study surveying clinicians (e.g., physicians and nurses) worldwide post-pandemic reported that 37% plan to leave their current roles in the next 2–3 years [41]. We suspect that attributes unique to the PA professions, such as career flexibility, may account for the high job satisfaction and lower burnout levels; however, more studies are needed to illuminate these findings.

Lastly, our study revealed that a higher proportion of PAs working in psychiatry are from a minority background and identify as women. Existing literature indicates that both women and minority psychiatrists are underrepresented in the psychiatry workforce [43]. Therefore, as PAs in psychiatry work alongside psychiatrists, the diverse workforce among psychiatry PAs could serve as a bridge to optimize access to mental health care for underrepresented minority individuals. Research suggests that patients adhere more to treatment plans when they share a similar background to their providers [44].

The rising demand for mental health services, the effects of the COVID-19 pandemic, and the scarcity of psychiatrists in the US are significant barriers to mental health services. Without increasing the presence of psychiatric and behavioral health provider positions, many regions will likely experience a continued supply shortage of psychiatric providers over the upcoming years. Expanding the mental health workforce to include PAs has advanced as a readily available human resource that already functions in behavioral health roles [25, 45, 46]. Several policies have likely contributed to the steady incline of the US PAs in psychiatry: the Affordable Care Act (ACA) of 2010, the Comprehensive Addiction and Recovery Act (CARA) of 2016, the Substance Use-Disorder Prevention that Promotes Opioid Recovery and Treatment (SUPPORT**)** of Patient and Communities Act of 2018, and the 2021 American Rescue Plan Act (ARPA) [47, 48]. These regulations are intended to expand access to care for individuals with mental health and substance abuse disorders. For instance, ACA aimed to provide more affordable health insurance and expand access to care; CARA aided in identifying the needs and services for individuals with mental health disorders and substance abuse, and SUPPORT assisted in providing adequate training to healthcare providers on medication-assisted treatment (MAT), including PAs and NPs. More recently, in 2021, the ARPA allocated $4 billion in grants to increase health services and enhance provider education to build a robust and diverse mental health/psychiatry workforce [48].

Likewise, PA national organizations and PA programs across the US have recognized the critical role of PAs in mental health and thus have instituted several initiatives to support the training of PAs in psychiatry. For instance, the nccPA Health Foundation board adopted mental health as a strategic goal area in 2015 to provide resources and community grants to promote “strategies and advance the role of PAs in addressing mental health” [49]. Concurrently, the development of postgraduate psychiatry/mental health clinical programs and Certificate of Added Qualifications (CAQs) in psychiatry are increasing to expand the skills and knowledge of PAs in this specialty to provide the highest quality of care for patients [50].

In summary, the characteristics of PAs in psychiatry, their distribution, and contribution serve as a foundation and reference for greater exploration of this PA role and activity. Future research efforts should focus on the influences and barriers PAs encounter pursuing a psychiatry clinical role, patient care outcomes, and productivity. Furthermore, the role and contribution of PAs globally in the mental health/psychiatry workforce should be further examined, as the prevalence of mental health disorders such as anxiety and depression likely doubled after the pandemic [51].

## Limitations

A notable limitation of our study is that it relied on self-report data, which may have been influenced by recall biases. Another limitation is the study’s design—only PAs who updated their profile for the past three years (2019–2021) were included. Thus, PAs who changed specialty (e.g., from other specialties to psychiatry, or vice versa) during the study period but did not update their profile could affect the study's results [52].

## Conclusion

Our findings demonstrate that the workforce of PAs in psychiatry is growing. This may be due to the response to the country’s needs—impacted by the scarcity of psychiatrists, the rise of mental health and substance use disorders, sequela from the COVID-19 pandemic, and initiatives and policies at the federal and state levels. Their employment represents a needed source of expertise in mental health delivery services. With this foundation of PAs in the psychiatry workforce, future research efforts should focus on the influences and barriers that PAs encounter pursuing a psychiatry clinical role, and targeted strategies should be implemented to address healthcare workforce disparities in psychiatry.

## Data Availability

The datasets generated and analyzed during the current study are not publicly available due to the confidentiality of individualized data, but de-identified data can be available if requested by the corresponding author.

## Abbreviations

AAMC: Association of American Medical Colleges
BLS: Bureau of Labor Statistics
ACA: Affordable Care Act
CARA: Comprehensive Addiction and Recovery Act
ARPA: American Rescue Plan Act
CAQs: Certificate of added qualifications
HRSA: Health Resources and Services Administration
MAT: Medication-assisted treatment
NCCPA: The National Commission on Certification of Physician Assistants
NIH: National Institute of Health
NP: Nurse practitioner
PA: Physician assistant or physician associate
SUPPORT: Substance Use-Disorder Prevention that Promotes Opioid Recovery and Treatment of Patient and Communities Act
URiM: Underrepresented in medicine
US: United States of America
VA: Veteran Affairs
WHO: The World Health Organization

## Footnotes

1 Nurse practitioners (NPs) are healthcare providers who complete advanced clinical training beyond their initial professional registered nurse preparation [8]. NPs generally specialize in several medical disciplines, such as family medicine, gerontology, mental health, pediatrics, and women’s health, while PA education is focused as generalists provider and are trained in the medical school education model [9] [10].
